# Supplementary material for: A Deletion in GDF7 is Associated with a Heritable Forebrain Commissural Malformation Concurrent with Ventriculomegaly and Interhemispheric Cysts in Cats
Source: Genes (Basel). 2020 Jun 19;11(6):672. doi: 10.3390/genes11060672 (PMC7349246; doi:10.3390/genes11060672)
Supplement: Supplementary file 1 [file genes-11-00672-s001.zip › genes-820548 supplementary/Supplementary/GDF7-FigS3.pdf]

```
Felis catus (wildtype)    MDLSAAAALC LWLLSACRPR DGLEAAAVLR AAGAGPVGSP GGDGGSGSGG RTLAPAAGVS 60
Felis catus (mutant)      .......... .......... .......... .......... .......... ..........
Homo sapiens              .......... .......... .......... .......R.. ..G..G.G.. ....Q...AA
Equus caballus            .......... .......... .......... ......A... ..--.G.G.. ....A...A.
Bos Taurus                .......... .......... .......... ......AE.. ..--.G.GS. T...A.E.T.
Rattus norvegicus         .......... .......... .......... ......AW.. ..----.G.. ....R.P.P.
Mus musculus              .......... .......... .......... ......AW.. ..----.G.. ......P.P.
Consensus                 MDLSAAAALC LWLLSACRPR DGLEAAAVLR AAGAGPAGSP GG--GGGGGG RTLAPAAGXS

Felis catus (wildtype)    AAPAAAAPGV RAARRAAGSG FRNGSVVPHQ FMMSLYRNLA GRAPAGAAAA STSGSGRHGR 120
Felis catus (mutant)      .......... ...PP.PA.. TARWCRTSS- ---------- ---------- ----------
Homo sapiens              .V....V.RA .......... .........H ......S... .........V .A..---...
Equus caballus            TG.......A .......S.. .......... ......S... .......... ..........
Bos Taurus                ..L...S..P .G....T... .......... ......S.T. ..T...PV.V ..........
Rattus norvegicus         .LQ...V..P ..V....... .........H ......S... ....V..... AA..---...
Mus musculus              .LQ...V..P ..V....... .........H ......S... .......... A...---...
Consensus                 AAPAAAXPGP RAARRAAGSG FRNGSVVPHX FMMSLYRSLA GRAPAGAAAA STSG---HGR

Felis catus (wildtype)    ADTITGFADQ ANQDESAA-E TGQSFLFDVS SLPDADEVVG AELRVLRRES PELGPGSATP 179
Felis catus (mutant)      ---------- ---------- ---------- ---------- ---------- ----------
Homo sapiens              .......T.. .T......-. .......... ..N....... ........G. ..S....W.S
Equus caballus            .......... .T......-. .......... ..S....... .........F ..PSS...I.
Bos Taurus                ...V...... .IP.....-Q ..LN...... ........L. .......... GAR.....S.
Rattus norvegicus         V......T.. .T...T..A. P......... ..SE.....N ........R. ..PDRD...L
Mus musculus              V......T.. .T.E....-. P......... ..S.S....N ........R. ..PDRD...L
Consensus                 ADTITGFXDQ ATQDESAA-E TGQSFLFDVS SLSDADEVVG AELRVLRRES PEPGPGSATP

Felis catus (wildtype)    LPLLLL-STC PGAARAPRLL HSRAAEPLAG ARWEVFDVAD AVRRHRQEPH ATRAFCLLLR 238
Felis catus (mutant)      ---------- ---------- ---------- ---------- ---------- ----------
Homo sapiens              P.....-... .......... Y.......V. Q...A..... .M....R..R PP........
Equus caballus            Q.....L... ....S..L.. ........D. .......... ......R..R ...V......
Bos Taurus                -.....-... .S..S..... ......F.DA .......... .L....R..R TS.....S.S
Rattus norvegicus         ..R...-... .DE.GTAH.. ........G. ....A...T. ..QS..RW.R .S.K...V..
Mus musculus              P.....-... .DE.GTAH.. ........DS ....A...T. ..QS..RW.R TS.K...V..
Consensus                 XPLLLL-STC PGAAXAPRLL HSRAAEPLDG ARWEXFDVAD AVRRHRREPR ASRAFCLLLR

Felis catus (wildtype)    SVAGPSQGPL ALRLLGFGSR GRD---GAAA EERALLVVSS RTQRKGSLFR EIRAQARALG 295
Felis catus (mutant)      ---------- ---------- ---------- ---------- ---------- ----------
Homo sapiens              A....VPS.. ...R....WP .GG---.S.. ....V..... .....E.... ..........
Equus caballus            AA...AR... ..Q....... .GG---.... .......... .....E.... ..........
Bos Taurus                G.V.SARV.. ...P....L. .GG---.... .......... .....E.... ...S......
Rattus norvegicus         A.TASESS.. ...R....WP .GGDGG.T.. .......I.. .....E.... .........R
Mus musculus              A.T.AESS.. ...R....WP .GGDGG.T.. .......I.. ..H..E.... .........R
Consensus                 AVAGPXXSPL ALRRLGFGWX GGG---GAAA EERALLVVSS RTQRKESLFR EIRAQARALG

Felis catus (wildtype)    AALAAEPPRD PGPGTGSPTA VISGRRRRRT ALTGARATQG SGGGGG---- ---------- 360
Felis catus (mutant)      ---------- ---------- ---------- ---------- ---------- ----------
Homo sapiens              ....S..LP. ..T..A..R. ..G....... ..A.T.TA.. ....------ ----------
Equus caballus            .P......P. .......... ..G....... ..A.T..A.. ....------ ----------
Bos Taurus                ....V.SRP. SR..V..... ..G....... ..A....A.. ....------ ----------
Rattus norvegicus         .--.....P. ....A..RK. NLG....... ..A.T.GA.. ......GGGG GGGGGGGGGG
Mus musculus              .--...L.P. ..L.A..RK. TPG....... ..A.T.GA.. ......GGGG GGGGGGG---
Consensus                 AALAAEPPPD PGPGTGSPTA VIGGRRRRRT ALAGTRAAQG SGGG------ ----------

Felis catus (wildtype)    -AGRGHGRRG RTRCSRRPLH VDFKELGWDD WIIAPLDYEA YHCEGVCDFP LRSHLEPTNH 400
Felis catus (mutant)      ---------- ---------- ---------- ---------- ---------- ----------
Homo sapiens              -......... .S....K... .......... .......... .....L.... ..........
Equus caballus            -......... .S....K... .......... .......... .......... ..........
Bos Taurus                -......... .S....K... .......... .......... .......... ..........
Rattus norvegicus         G......... .S....KS.. .......... .......... .......... ..........
Mus musculus              -......... .S....K... .......... .......... .......... ..........
Consensus                 -AGRGHGRRG RSRCSRKPLH VDFKELGWDD WIIAPLDYEA YHCEGVCDFP LRSHLEPTNH
```

Felis catus (wildtype)	AIIQTLLNSM	APDAAPASCC	VPARLSPISI	LYIDAANNVV	YKQYEDMVVE	ACGCR	455
Felis catus (mutant)	----------	----------	----------	----------	----------	-----	
Homo sapiens	....	....	....	....	....	.....	
Equus caballus	....	....	....	....	....	.....	
Bos Taurus	....	....	....	....	....	.....	
Rattus norvegicus	....	....	....	....	....	.....	
Mus musculus	....	....	....	....	....	.....	
Consensus	AIIQTLLNSM	APDAAPASCC	VPARLSPISI	LYIDAANNVV	YKQYEDMVVE	ACGCR	
