# Supplementary material for: A Deletion in GDF7 is Associated with a Heritable Forebrain Commissural Malformation Concurrent with Ventriculomegaly and Interhemispheric Cysts in Cats
Source: Genes (Basel). 2020 Jun 19;11(6):672. doi: 10.3390/genes11060672 (PMC7349246; doi:10.3390/genes11060672)
Supplement: Supplementary file 1 [file genes-11-00672-s001.zip › genes-820548 supplementary/Supplementary/GDF7-Table S1-FINAL-18June.pdf]

Table S1. Regions of homozygosity that was unique to 18 cats with the inherited forebrain commissural malformation, and were absent in all the unaffected cats.

SNP*	Chr. A3 Position†
chrA3.160566453	125601560
chrA3.160530122	125625991
chrUn.24474142	125637518
chrUn.47705095	125688296
chrA3.160408014	125732886
chrA3.160379824	125762335
chrA3.160343100	125791293
chrA3.160337238	125795275
chrA3.160313108	125815756
chrA3.160271820	125839780
chrA3.160202681	125898946
chrA3.160122222	125972504
chrA3.160090785	125992080
chrA3.160044067	126031109
chrA3.160043010	126032117
chrA3.160002944	126063095
chrA3.159964768	126096458
chrA3.159917387	126130456
chrA3.159873847	126155695
chrA3.159846599	126189378
chrA3.159815186	126219694
chrA3.159670655	126341357
chrA3.159646777	126359090
chrA3.159621145	126377299
chrA3.159565119	126419390
chrA3.159538209	126438891
chrA3.159462631	126500287
chrA3.159427910	126532359
chrA3.159396654	126546963
chrA3.159339796	126592318
chrA3.159285393	126633822
chrA3.159248736	126665749
chrA3.159136100	126756640
chrA3.159105911	126769774
chrA3.159072838	126802789
chrA3.158996855	126872329
chrA3.158967674	126896343

chrA3.158926828	126925447
chrA3.158896738	126953595
chrA3.158828000	127003756
chrA3.158754772	127069181
chrA3.158724720	127099328
chrA3.158673277	127142715
chrA3.158624618	127189752
chrA3.158518903	127274926
chrA3.158488087	127298681
chrA3.158461077	127325195
chrA3.158433403	127348216
chrA3.158389360	127386120
chrA3.158358606	127398951
chrA3.158279342	127468992
chrA3.158214221	127531499
chrA3.158123659	127601646
chrA3.158064272	127654673
chrA3.158029141	127684693

*SNP IDs are based on an early cat genome assembly [26] †Positions based on current cat genome assembly [27].
