# Supplementary figures and images for: A Deletion in GDF7 is Associated with a Heritable Forebrain Commissural Malformation Concurrent with Ventriculomegaly and Interhemispheric Cysts in Cats
Source: Genes (Basel). 2020 Jun 19;11(6):672. doi: 10.3390/genes11060672 (PMC7349246; doi:10.3390/genes11060672)

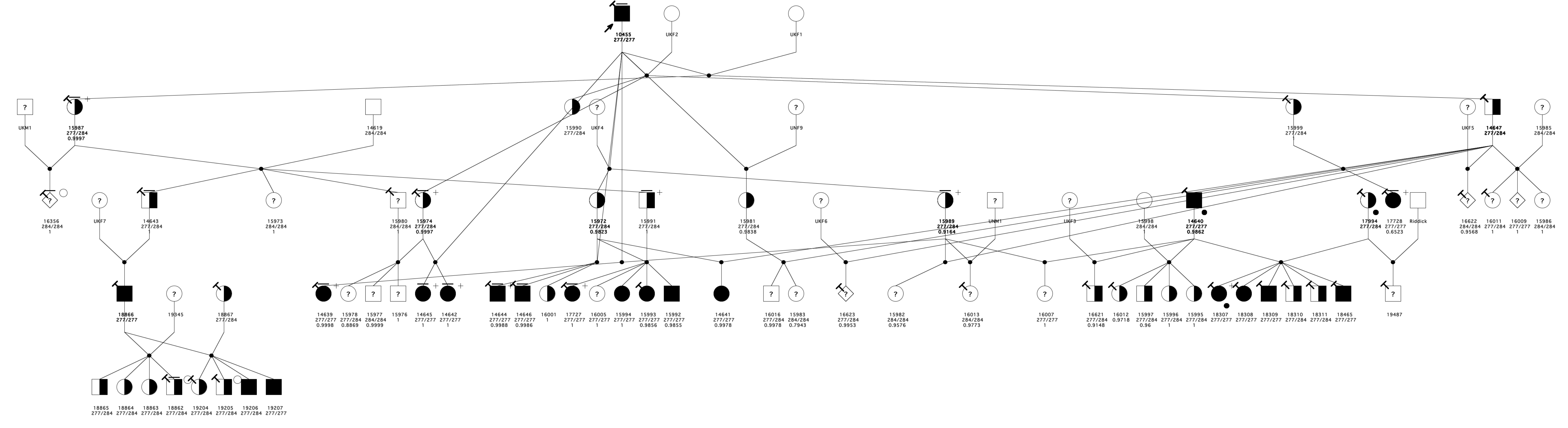

Supplement: Supplementary file 1 [file genes-11-00672-s001.zip › genes-820548 supplementary/Supplementary/GDF7-FigS1.tif]

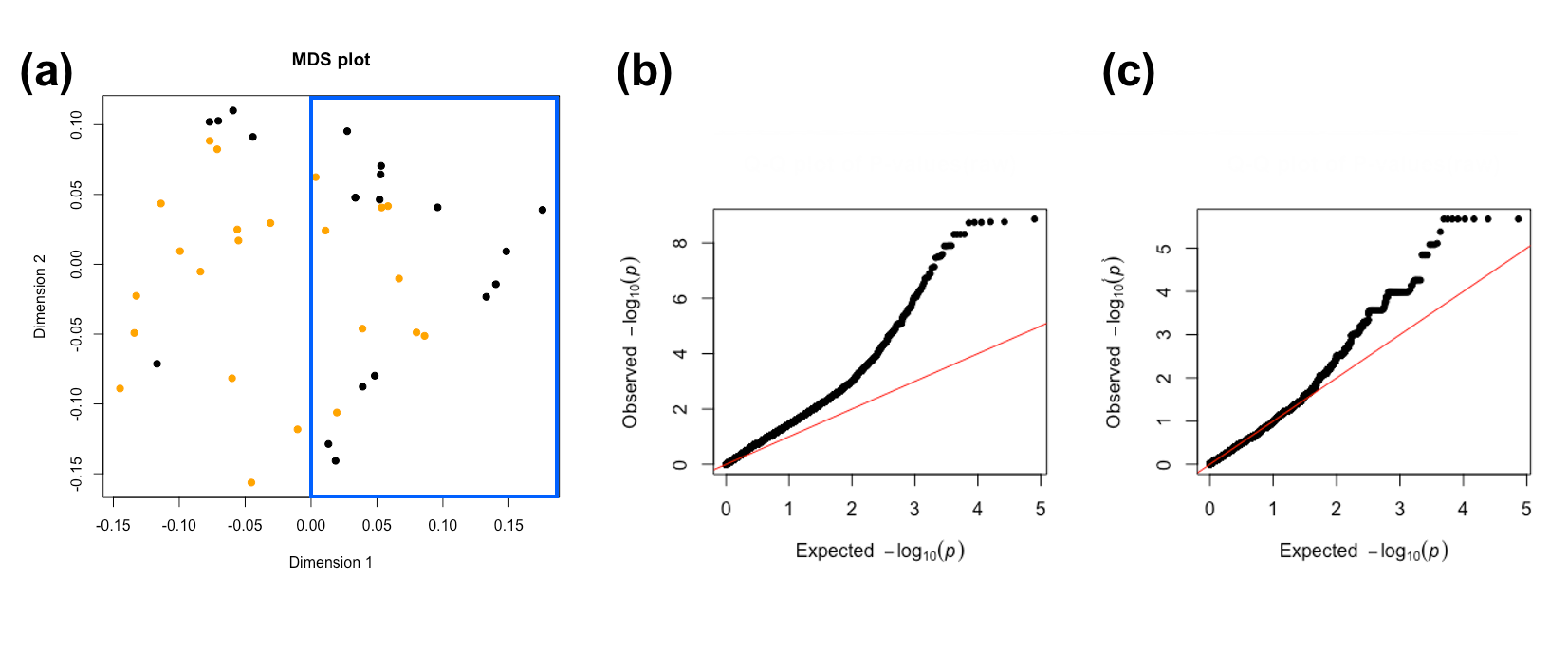

Supplement: Supplementary file 1 [file genes-11-00672-s001.zip › genes-820548 supplementary/Supplementary/GDF7-FigS2.tiff]

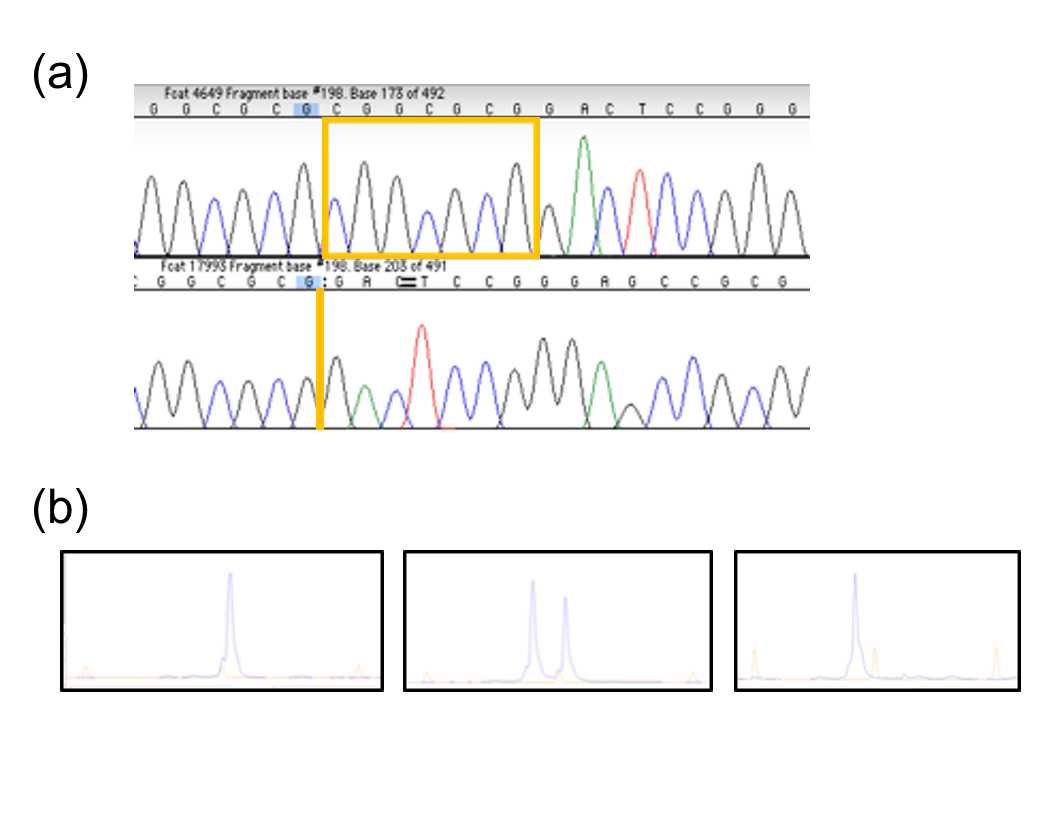

Supplement: Supplementary file 1 [file genes-11-00672-s001.zip › genes-820548 supplementary/Supplementary/GDF7-FigS4.tiff]
